# RETRACTED: Abu Bakar et al. Celastrol Protects Against Antimycin A-Induced Insulin Resistance in Human Skeletal Muscle Cells. *Molecules* 2015, *20*, 8242–8269

**DOI:** 10.3390/molecules31152576

**Published:** 2026-07-24

**Authors:** Mohamad Hafizi Abu Bakar, Kian-Kai Cheng, Mohamad Roji Sarmidi, Harisun Yaakob, Hasniza Zaman Huri

**Affiliations:** 1 Department of Bioprocess Engineering, Faculty of Chemical Engineering, Universiti Teknologi Malaysia, Johor Bahru 81310, Johor, Malaysia; chengkiankai@cheme.utm.my; 2 Innovation Centre in Agritechnology for Advanced Bioprocessing (ICA), Universiti Teknologi Malaysia, Skudai 81310, Johor, Malaysia; 3 Institute of Bioproduct Development, Universiti Teknologi Malaysia, Johor Bahru 81310, Johor, Malaysia; 4 Department of Pharmacy, Faculty of Medicine, University of Malaya, Kuala Lumpur 50603, Malaysia; hasnizazh@ummc.edu.my; 5 Clinical Investigation Centre, 13th Floor Main Tower, University Malaya Medical Centre, Lembah Pantai 59100, Kuala Lumpur, Malaysia

The journal retracts the article titled “Celastrol Protects Against Antimycin A-induced Insulin Resistance in Human Skeletal Muscle Cells” [1], cited above.

Following publication, concerns were brought to the attention of the Editorial Office regarding the integrity of figures presented in this article [1].

In accordance with the journals’ procedures, the Editorial Office and Editorial Board conducted an investigation that identified indicators of inappropriate editing and duplication in panels presented in Figure 5, Figure 7 and Figure 8, including material overlapping with that presented in a previously published article [2]. Although the authors cooperated with the investigation, the explanations provided were not deemed sufficient by the Editorial Board to resolve the identified concerns.

As a consequence, the Editorial Board has lost confidence in the reliability of the reported findings and has decided to retract this publication in accordance with MDPI’s retraction policy. This retraction was approved by the Editor-in-Chief of *Molecules*.

The authors have been informed of this retraction but did not provide a comment on this decision.
